# The complete mitochondrial genome of the freshwater snail *Cipangopaludina ampullacea* (Küster, 1852) (Gastropoda: Viviparidae)

**DOI:** 10.1080/23802359.2022.2116945

**Published:** 2022-09-05

**Authors:** Meng-Meng Wu, Hui-Zhong Cheng, Li-Li Li, Guang-Long Xie

**Affiliations:** a Shandong Freshwater Fisheries Research Institute, Jinan, P.R. China; b School of Life Sciences, Qufu Normal University, Qufu, P.R. China

**Keywords:** Caenogastropoda, Viviparoidea, phylogenetics, China

## Abstract

*Cipangopaludina ampullacea* (Küster, 1852) is a freshwater snail endemic to China. In this study, the complete mitochondrial genome of *C. ampullacea* was sequenced using next-generation sequencing. The mitogenome is 16,892 bp long and comprises a total of 37 genes, including 13 protein-coding genes, two rRNA genes, and 22 tRNA genes. It is consistent with the basic characteristics of other known viviparid mitochondrial genomes. Phylogenetic analysis using related species mitogenomes showed that *Cipangopaludina* and *Margarya* are mutually non monophyletic. Our study provides valuable information to reconstruct the taxonomy and evolution of viviparid snails more comprehensively.

Viviparidae is a family of rather large freshwater gastropods, containing approximately 150 species (Franke et al. 2007). Except for South America and Antarctica, this family is represented on every continent, where it inhabits areas such as lakes, ponds, and rivers (Strong et al. 2008). Due to the incongruence among the morphology and molecular phylogeny of the Viviparidae, the classification of this family is still ambiguous, especially within the genus *Cipangopaludina* (Wang et al. 2017; Stelbrink et al. 2020). *Cipangopaludina ampullacea* (Küster 1852) is a viviparid snail endemic to China, mainly distributed in Yunnan and Sichuan provinces (Liu et al. 1995). Due to the decreasing of its natural populations, *C. ampullacea* is currently listed as Vulnerable (VU) by the Chinese Species Red List (Vol. III Invertebrates). Studies about the shell morphology of *C. ampullaceal* are still scarce (Liu et al. 1995; Lu et al. 2014) and only very few mitochondrial genomes from the genus *Cipangopaludina* have been published so far (Wang et al. 2017; Nasu et al. 2020). In this study, we sequenced the complete mitochondrial genome of *C. ampullacea* as a resource for promoting phylogenetic studies of the Viviparidae. Our study provides valuable data that can be used in the taxonomy and evolution of viviparid snails.

Specimens of *C. ampullacea* were collected from Lake Dianchi (24°47′11″N, 102°36'52″E), Yunnan, China. Total genomic DNA was extracted using EZNA Mollusk DNA Kit (Omega Bio-Tek, USA) according to the manufacturer’s instructions. The muscle tissue was preserved at −80 °C, and the voucher specimen (number: 315-VIVI-QFNU; contact Guang-Long Xie: gxie@hotmail.com) was deposited in the Zoology Museum of Qufu Normal University. The mitochondrial genome of *C. ampullacea* was sequenced on the Illumina Novaseq 6000 sequencing platform with PE150 strategy. The sequence was assembled and mapped within CLC Genomic Workbench 12.0.2 (Qiagen). The mitochondrial genome was annotated using the MITOS webserver (Bernt et al. 2013). The preliminary results were compared with the protein-coding genes (PCGs) and rRNA genes of other viviparid species by using BLAST searches. MITOS and ARWEN were used to detect tRNA genes (Laslett and Canback 2008). Phylogenetic relationships of Viviparidae were reconstructed by performing Maximum Likelihood (ML) and Bayesian inference (BI). For Maximum Likelihood analyses were inferred by using IQ-Tree, nodal support of the best tree was estimated by performing 10,000 ultrafast bootstrap replicates (Nguyen et al. 2015). For the Bayesian analyses were inferred by MrBayes version 3.2.6 (Ronquist et al. 2012). Bayesian posterior probabilities of phylogenetic trees were estimated by running four separate runs of each 10 million generations. Each run had four chains, of which one was heated. Sampling rate was every 1000 generations.

The complete mitogenome of *C. ampullacea* (GenBank accession number: MZ488942) was 16,891 bp in length. Identical to other Viviparidae species, it contains 13 protein-coding genes (PCGs), 22 transfer RNA (tRNAs), and two ribosomal RNA (rRNAs). The base composition of the whole heavy strand is A 27.2%, C 8.2%, G 20.1%, T 44.5%. The A + T content (71.7%) was distinctly higher than the GC content (28.3%). Thirty genes were located on the heavy strand while all others were located on the light strand (seven tRNA genes: *trnY, trnC, trnW, trnM, trnQ, trnG,* and *trnE,*). The gene arrangement of *C. ampullacea* differed slightly from that of other known viviparid mitogenomes, in the translocation on the relative position of the *YCWMQGE* tRNA clusters (Wang et al. 2017).

Together with 17 other viviparid species, phylogenetic analysis based on 13 PCGs genes showed that the phylogenetic status of *C. ampullacea* differed in the BI and ML trees (Figure 1). In the ML tree, *C. ampullacea* was the sister group with *Margarya melanioides.* By contrast, in the BI tree, the *C. ampullacea was the sister group with M. oxytropoides* + *M. melanioides*. Moreover, *Cipangopaludina* and *Margarya* were non-monophyletic, which is consistent with previous study. The phylogenetic relationships of *Cipangopaludina* and *Margarya* were controversial (Wang et al. 2017; Stelbrink et al. 2020). Hence, these genera need integrative taxonomic revisions.

**Figure 1.**
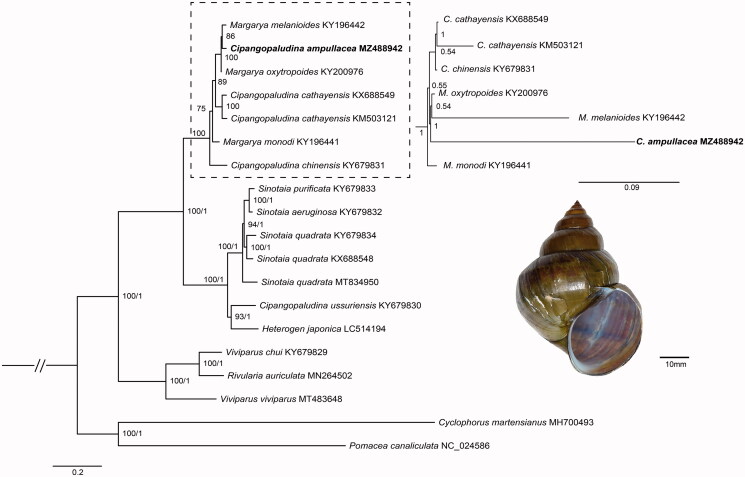
Maximum likelihood tree of 17 viviparid gastropods based on 13 PCGs of mitogenomes. *Pomacea canaliculata* (Ampullariidae), and *Cyclophorus martensianus* (Cyclophoridae) were used as outgroup taxa.

## Data Availability

The genome sequence data that support the findings of this study are openly available in GenBank of NCBI at (https://www.ncbi.nlm.nih.gov/) under accession no MZ488942. The associated BioProject, SRA, and Bio-Sample numbers are PRJNA770541, SRR16304169, and SAMN22224088 respectively.
